# Trends in incidence of anal cancer in Austria, 1983–2016

**DOI:** 10.1007/s00508-020-01622-z

**Published:** 2020-03-04

**Authors:** Emily Heer, Monika Hackl, Monika Ferlitsch, Thomas Waldhoer, Lin Yang

**Affiliations:** 1 Cancer Epidemiology and Prevention Research, Alberta Health Services, Calgary, Canada; 2 Austrian National Cancer Registry, Statistics Austria, Vienna, Austria; 3 Department of Internal Medicine III, Division of Gastroenterology and Hepatology, Medical University of Vienna, Vienna, Austria; 4 Department of Epidemiology, Center for Public Health, Medical University of Vienna, Kinderspitalgasse 15, Vienna, Austria; 5 Department of Oncology & Community Health Sciences, Cumming School of Medicine, University of Calgary, Calgary, Canada

**Keywords:** Anal cancer, Time trend, Austria, Screening, Incidence

## Abstract

**Background:**

Recent reports have noted increasing rates of anal cancer among high-income countries worldwide; however, little is known about these trends in Austria.

**Methods:**

Data on anal cancer from 1983 to 2016 were obtained from Statistics Austria. All tumors (*n* = 3567) were classified into anal squamous cell carcinomas (ASCC), anal adenocarcinomas (AADC), and others (unspecified carcinoma and other specific carcinoma). Anal cancer incidence rates were calculated in 5‑year cycles and incidence average annual percentage change (AAPC) to evaluate trends by sex, histology and age group.

**Results:**

The incidence rate of anal cancer was higher among females than males (relative risk, RR = 1.66, 95% confidence interval, CI: 1.55–1.79, *p* < 0.0001). From 1983 through 2016, incident anal cancer increased significantly (0.92 per 100,000 person-years to 1.85 per 100,000 person-years, AAPC = 1.93, 95% CI: 1.52 to 2.34, *p* < 0.0001), particularly among those 40–69 years old. From 1983 through 2016, the increasing anal cancer incidence was primarily driven by ASCC (0.47–1.20 per 100,000 person-years, AAPC = 2.23, 95% CI: 1.58 to 2.88, *p* < 0.0001) and others (other than ASCC and AADC, AAPC = 1.78, 95% CI: 1.01–2.55), yet stable in AADC (AAPC = 0.88, 95% CI: −0.48–2.25).

**Conclusions:**

Despite being a rare cancer in Austria, the increase in anal cancer incidence rate from 1983 to 2016 was substantial, particularly in ASCC. The observed rising trends reflect the need to investigate associated risk factors that have increased over time to inform preventive measures.

**Electronic supplementary material:**

The online version of this article (10.1007/s00508-020-01622-z) contains supplementary material, which is available to authorized users.

## Introduction

Anal cancer is a relatively uncommon malignancy, accounting for approximately 4% of all cancers of the lower gastrointestinal tract [1]. Worldwide incidence of this cancer is 1–2 per 100,000 person-years in average risk groups, with the incidence being slightly higher for women compared to men [2, 3]. Although rare, reports have shown that incidence has increased in many high-income countries, including Canada, the United States of America [4], Denmark [5], and the United Kingdom [6, 7]. These increases are largely occurring for anal squamous cell carcinomas (ASCC), with smaller changes or decreases for adenocarcinomas (AADC), and the reasons are not entirely clear [2, 8]. Anal cancer has a relatively high mortality rate, with a 5-year survival rate among those diagnosed with stage 4 of below 50% [9]. Therefore, preventing further increases in incidence is imperative to reduce further morbidity and mortality.

The majority of ASCC cases are considered to be caused by human papillomavirus (HPV) infection [10], and risk factors include number of sexual partners, receptive anal intercourse, smoking [8, 10], history of sexually transmitted infections [11], and immune deficiency, i.e. infection with human immunodeficiency virus (HIV) or immunosuppressive drugs [8]. These risk factors are similar for cervical cancer, another malignancy caused by HPV infection [12].

Epidemiological data on anal cancer in Austria is sparse; one published report gives the number of cases between 2008 and 2012 at 230 and 454 for men and women, respectively [12], but the contemporary trends of anal cancer incidence in Austria are unknown. Using the most recent and updated information on anal cancer incidence (1983–2016) from Statistics Austria, the objective of the present analysis was to systematically describe the national cancer incidence rates over more than 30 years.

## Methods

Anal cancer cases were defined according to the International Classification of Diseases version (ICD)-10, with code C21 identified as anal cancer from the Austrian Cancer Registry (derived on 19 December 2018). For histological classification, all tumors were classified into three categories based on ICD oncology version 3 (ICD-O-3) codes, per World Health Organization (WHO) category conventions [13]: anal squamous cell carcinoma (ASCC, 8050–8076); anal adenocarcinomas (AADC), and “other” (unspecified carcinoma 8010-8034 and other specified carcinoma, all remaining morphology codes). Age groups were defined in 10-year intervals starting at age 30 years (<30, 30–39, 40–49, 50–59, 60–69, 70–79, 80+ years).

### Statistical analyses

Age-specific incidence rates for anal cancer were calculated in 5‑year cycles to obtain a stable estimation (the last cycle contains only 4 years: 2013–2016). Age standardized rates were calculated by year and sex using the European standard population [14].

Average annual percent changes (AAPCs) in incidence were calculated to describe trends from 1983 to 2017. In addition, APCs were described by sex, histology, and age group to identify trends by subgroups. Comparison of crude incidence rates between the years 1983–1987 and 2013–2016 by age and sex was done by the exact Fisher test using SAS version 9.4 (SAS Institute, Cary NC, USA). Time trend analysis and calculation of adjusted incidence rates were done by Joinpoint software (Joinpoint Regression Program, Version 4.7.0.0. February, 2019; Statistical Research and Applications Branch, National Cancer Institute, Bethesda, Maryland, USA). The maximum number of joinpoints was set to two, the minimum number to either side of the end and between joinpoints to two, respectively. A *p*-value <0.05 was considered to be statistically significant. No adjustment for multiple testing was done therefore *p*-values are to be interpreted exploratorily only. This study does not require an approval by an institutional review board because only publicly accessible records were used without any contact with individuals.

## Results

**Fig. 1:**
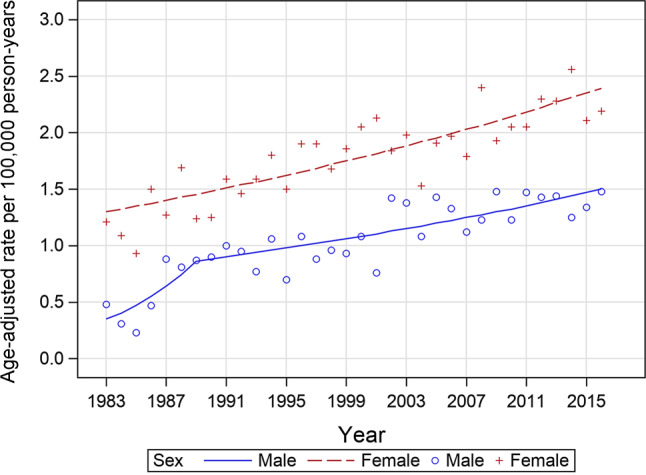
Age-adjusted incidence rates per 100,000 person-years and trends of anal cancer in Austria in males and females from 1983 through 2016

Between 1983 and 2016, a total of 1099 and 2468 cases of anal cancers were identified in males and females, respectively (Table 1). Overall, anal cancer incidence rates increased significantly from 1983 (0.92 per 100,000 person-years) through 2016 (7.84 per 100,000 person-years), with an APC of 1.93 (95% CI 1.5–2.3) (Fig. 1, Supplementary Table 1). The incidence rates of anal cancer were consistently higher among females compared with males (odds ratio, OR 1.66, 95% CI 1.55–1.79), and exhibited increases in both sexes over time (male: 0.35 per 100,000 person-years to 1.50 per 100,000 person-years, APC 1983–1989 of 16.5, 95% CI 2.8–32.0, APC 1989–2016 of 2.0, 95% CI 1.3–2.8; female: 1.30 per 100,000 person-years to 2.39 per 100,000 person-years, APC of 1.9, 95% CI 1.4–2.3).

**Fig. 2:**
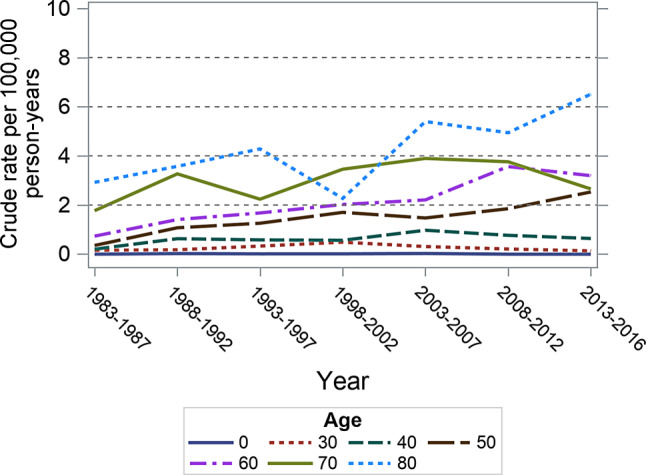
Age-specific anal cancer incidence rates in male Austrian residents from 1983 through 2016

**Fig. 3:**
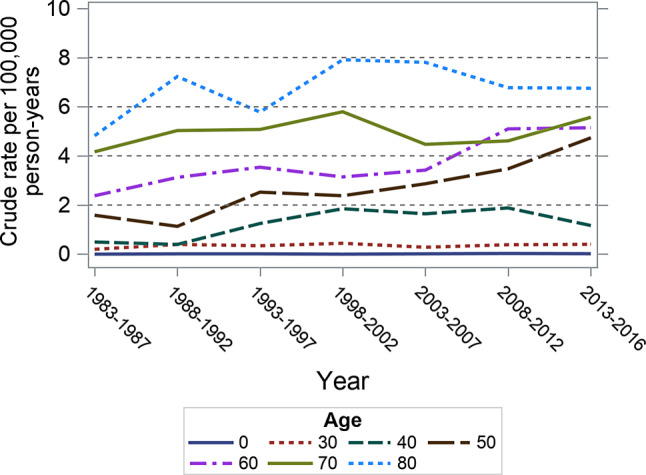
Age-specific anal cancer incidence rates in female Austrian residents from 1983 through 2016

In subgroups by age, the increase in anal cancer incidence rates was particularly evident among those aged 40–69 years (Figs. 2 and 3, Supplementary Table 2). In the male population, anal cancer incidence rate increased from 0.20 (95% CI 0.03–0.38) to 0.64 (95% CI 0.34–0.94) among 40–49 years age band, increased from 0.35 (95% CI 0.09–0.62) to 2.53 (95% CI 1.91–3.16) among 50–59 years age band, and increased from 0.73 (95% CI 0.28–1.19) to 3.20 (95% CI 2.35–4.04) among 60–69 years age band. In the female population, these increases were from 0.49 (95% CI 0.21–0.77) to 1.17 (95% CI 0.76–1.58) among 40–49 years age band, 1.58 (1.05–2.12) to 4.74 (3.88–5.59) among 50–59 age band, and from 2.38 (95% CI 1.72–3.04) to 5.15 (95% CI 4.12–6.17) among 60–69 years age band. The incidence rates of anal cancer rose in males and females. The increases in each age group appear to be similar, except a significant age and sex interaction (*p* = 0.03) observed among those 60–69 years old with the annual increase in males exceeding that of females.

Across sub-types of anal cancer, the increasing anal cancer incidence was primarily driven by ASCC (APC 2.23, 95% CI 1.58–2.88) and “others” (neither ASCC nor AADC) (APC 1.78, 95% CI 1.01–2.55), while incidence was stable for AADC (APC 0.88, 95% CI −0.48–2.25).

## Discussion

These analyses examined anal cancer incidence trends over more than 30 years in Austria. These trends have not been previously detailed and add further evidence for a global trend of increasing anal cancer rates. Between 1983 and 2016, the age-standardized rate of anal cancer was found to increase by an average of 1.93% per year, with particular increases among those aged 40–69 years. Particularly, the increasing rate of ASCC was driving the overall increase in incidence, while AADC incidence remained relatively stable. In addition, females were more likely to be diagnosed with anal cancer and exhibited higher annual percent changes than males.

These results are in line with other reports from high-income countries. In Australia, incidence of ASCC increased linearly by an APC of 1.6%, with recent decreases in AADC [15]. Larger increases were noted in France, where incidence of ASCC among women increased by 3.4% between 2005 and 2012, and 2.6% among men during the same time period [16]. Changes in incidence in the USA were similar to those in France, with ASCC increasing by 2.9%, and non-significant decreases in AADC [4].

The reasons for the observed trends are speculative, but likely reflect changing behavior related to known risk factors. As mentioned previously, anal HPV infection is believed to be a necessary factor in the development of ASCC, as almost all cases test positive for HPV DNA [17]. The risk of HPV increases with higher number of male sexual partners and receptive anal intercourse [11]. Today, people are more likely to have extramarital sex and have more sexual partners than people in the 1970s, and the prevalence of anal intercourse among heterosexual couples has increased [18]. These practices may have resulted in an increase in population HPV infection, and a subsequent increase in anal cancer incidence.

Another important risk factor for anal cancer is infection with HIV. A meta-analysis from North America found that the incidence ratio of anal cancer comparing those with HIV to those without was 28, and the incidence was as high as 131 per 100,000 individuals among men living with HIV [19]. While the incidence and mortality of HIV infection has decreased dramatically since the introduction of antiretroviral therapies (ART) [20], ART may not reduce the risk of anal cancer among those already with the infection [21]. In fact, longer time living with HIV is associated with increased risk of the cancer [22], unless treatment is initiated early [23]. Despite significant decreases in HIV incidence, people living with this infection are a high-risk group for anal cancer and should remain a priority for both primary and secondary prevention.

It is possible that these increasing trends may reverse when younger generations reach an age when anal cancer is usually diagnosed. The widespread use of HPV vaccination [9], participation in screening programs [24], and a changing sexual culture [25] may contribute to a reduction in anal cancer incidence in the future. Additionally, the reductions in new HIV infections [20] and global reductions in tobacco smoking [26] may also place younger generations at lower risk for anal cancer. Primary prevention with the HPV vaccine will continue to be important in reducing the incidence of anal cancer. In Austria, routine vaccination began in 2014 for boys and girls starting at the age of 9 years, but vaccination coverage is not currently known [12]. In the USA, the Advisory Committee on Immunization Practices recommended use of the quadrivalent HPV vaccine for prevention of anal cancer [27]. This vaccine was shown to protect against precancerous anal epithelial cells, particularly among high-risk groups, and risk of persistent infection was dramatically reduced [28]. These results show that HPV vaccination is an important tool in the prevention of anal cancer development, and higher rates of uptake are required.

Organized screening programs using Pap-tests for cervical cancer are widespread; coverage with Austria’s program ranges from 70–76% depending on the region [12]. While there is no established program for anal cancer screening, results from a recent pooled report suggest that data collected at cervical cancer screening can be used to identify women who should be screened for anal cancer [29]. In this study, 41% of immunocompetent women with cervical HPV-16 also had anal HPV-16, compared to only 2% who did not have the cervical strain. Therefore, high-risk cervical HPV appears to be a determinant for anal cancer risk, and routine cervical cancer screening could be used as a prevention tool for anal cancer as well [24].

Another possible reason for the increase of incidence of anal cancer is implementation of colorectal cancer screening programs, because the anus is also investigated during colonoscopy [30]. In Austria, an opportunistic colorectal screening program with primary screening colonoscopy was implemented in 2015. Therefore, the rise in anal cancer diagnosis in the last decade might be due to implementation of colorectal cancer screening programs.

These results, showing increasing anal cancer incidence in Austria, provide further evidence of a broader trend among high-income countries. Although rare, anal cancer has a relatively high mortality rate, and may have long-lasting health consequences for those who survive the disease [31]. Despite these alarming observations, anal cancer benefits from being a preventable disease in most cases. Widespread HPV vaccination among boys and girls, as well as comprehensive sexual education, including information about screening for HPV-related diseases may be critical in reducing incidence. Additionally, anal cancer and other HPV-related cancers should continue to be a focus of care among high-risk groups, including those living with HIV. Further research should also be conducted on determinants of progression from anal HPV to cancer, and possible biomarkers associated with progression. Trends in anal cancer should be continually monitored to identify any other target groups that may warrant closer surveillance or prevention efforts.

### Key points

- Between 1983 and 2016 the age-standardized rate of anal cancer increased by an average of nearly 2% per year.
- Females were more likely to be diagnosed with anal cancer and exhibited higher annual percent changes than males.
- The increasing anal cancer incidence was primarily driven by anal squamous cell carcinomas and “others” but stable in adenocarcinomas.

**Table 1:** Sex-specific numbers of cases and age-standardized incidence rates of anal cancer from 1983–1987 to 2013–2016 in Austria

Age (yrs)	1983–1987		2013–2016		*p-* value
	Cases	Incidence (95% CI)	Cases	Incidence (95% CI)	
*Male*					
<30	0	0.00 (0.00–0.00)	0	0.00 (0.00–0.00)	–
30–39	4	0.16 (0.00–0.31)	3	0.13 (0.00–0.28)	0.9999
40–49	5	0.20 (0.03–0.38)	17	0.64 (0.34–0.94)	0.0191
50–59	7	0.35 (0.09–0.62)	63	2.53 (1.91–3.16)	<0.0001
60–69	10	0.73 (0.28–1.19)	55	3.20 (2.35–4.04)	<0.0001
70–79	18	1.77 (0.95–2.59)	34	2.66 (1.76–3.55)	0.2085
80+	10	2.92 (1.11–4.74)	38	6.51 (4.44–8.58)	0.0239
*Female*					
<30	0	0.00 (0.00–0.00)	1	0.02 (0.00–0.05)	0.2280
30–39	5	0.20 (0.02–0.37)	9	0.40 (0.14–0.66)	0.2844
40–49	12	0.49 (0.21–0.77)	31	1.17 (0.76–1.58)	0.0093
50–59	34	1.58 (1.05–2.12)	119	4.74 (3.88–5.59)	<0.0001
60–69	50	2.38 (1.72–3.04)	97	5.15 (4.12–6.17)	<0.0001
70–79	76	4.17 (3.23–5.11)	88	5.57 (4.41–6.74)	0.0715
80+	41	4.82 (3.35–6.30)	76	6.76 (5.24–8.27)	0.0925

## Caption Electronic Supplementary Material

Supplemental Table 1: Sex-specific age-standardised incidence rates of anal cancer from 1983 through 2016 in Austria; Supplemental Table 2. Sex-specific crude incidence rates of anal cancer from 1983–1987 through 2013–2016 in Austria
