# Supplementary material for: Trends in incidence of anal cancer in Austria, 1983–2016
Source: Wien Klin Wochenschr. 2020 Mar 4;132(15):438–43. doi: 10.1007/s00508-020-01622-z (PMC7445201; doi:10.1007/s00508-020-01622-z)
Supplement: Supplementary file 1 — Supplemental Table 1: Sex-specific age-standardised incidence rates of anal cancer from 1983 through 2016 in Austria; Supplemental Table 2. Sex-specific crude incidence rates of anal cancer from 1983–1987 through 2013–2016 in Austria [file 508_2020_1622_MOESM1_ESM.docx]

| **Supplemental Table 1. Sex-specific age-standardised incidence rates of anal cancer (/100000) from 1983 through 2016 in Austria** | | | | | |
| --- | --- | --- | --- | --- | --- |
|  | Male |  |  | Female |  |
| 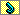Year | Incidence rate | SE |  | Incidence rate | SE |
| 1983 | 0.48 | 0.14 |  | 1.21 | 0.19 |
| 1984 | 0.31 | 0.12 |  | 1.09 | 0.17 |
| 1985 | 0.23 | 0.1 |  | 0.93 | 0.17 |
| 1986 | 0.47 | 0.14 |  | 1.5 | 0.2 |
| 1987 | 0.88 | 0.28 |  | 1.27 | 0.2 |
| 1988 | 0.81 | 0.21 |  | 1.69 | 0.21 |
| 1989 | 0.87 | 0.26 |  | 1.24 | 0.19 |
| 1990 | 0.9 | 0.19 |  | 1.25 | 0.19 |
| 1991 | 1 | 0.21 |  | 1.59 | 0.21 |
| 1992 | 0.95 | 0.2 |  | 1.46 | 0.2 |
| 1993 | 0.77 | 0.18 |  | 1.59 | 0.21 |
| 1994 | 1.06 | 0.21 |  | 1.8 | 0.23 |
| 1995 | 0.7 | 0.16 |  | 1.5 | 0.2 |
| 1996 | 1.08 | 0.23 |  | 1.9 | 0.23 |
| 1997 | 0.88 | 0.18 |  | 1.9 | 0.22 |
| 1998 | 0.96 | 0.19 |  | 1.68 | 0.21 |
| 1999 | 0.93 | 0.18 |  | 1.86 | 0.22 |
| 2000 | 1.08 | 0.19 |  | 2.05 | 0.23 |
| 2001 | 0.76 | 0.16 |  | 2.13 | 0.23 |
| 2002 | 1.42 | 0.22 |  | 1.84 | 0.21 |
| 2003 | 1.38 | 0.23 |  | 1.98 | 0.22 |
| 2004 | 1.08 | 0.2 |  | 1.53 | 0.19 |
| 2005 | 1.43 | 0.25 |  | 1.91 | 0.21 |
| 2006 | 1.33 | 0.21 |  | 1.97 | 0.22 |
| 2007 | 1.12 | 0.18 |  | 1.79 | 0.21 |
| 2008 | 1.23 | 0.19 |  | 2.4 | 0.24 |
| 2009 | 1.48 | 0.21 |  | 1.93 | 0.21 |
| 2010 | 1.23 | 0.21 |  | 2.05 | 0.22 |
| 2011 | 1.47 | 0.2 |  | 2.05 | 0.22 |
| 2012 | 1.43 | 0.2 |  | 2.3 | 0.23 |
| 2013 | 1.44 | 0.22 |  | 2.28 | 0.23 |
| 2014 | 1.25 | 0.19 |  | 2.56 | 0.24 |
| 2015 | 1.34 | 0.18 |  | 2.11 | 0.21 |
| 2016 | 1.48 | 0.2 |  | 2.19 | 0.22 |

SE: Standard error

| **Supplemental Table 2. Sex-specific crude incidence rates of anal cancer (/100000) from 1983-1987 through 2013-2016 in Austria** | | | | | | | | | | | | | | | | | | | | | |
| --- | --- | --- | --- | --- | --- | --- | --- | --- | --- | --- | --- | --- | --- | --- | --- | --- | --- | --- | --- | --- | --- |
|  |  | 1983-1987 | |  | 1988-1992 | |  | 1993-1997 | |  | 1998-2002 | |  | 2003-2007 | |  | 2008-2012 | |  | 2013-2016 | |
|  | Age | Incidence rate | SE |  | Crude Incidence | SE |  | Crude Incidence | SE |  | Crude Incidence | SE |  | Crude Incidence | SE |  | Crude Incidence | SE |  | Crude Incidence | SE |
| Male | 0-<30 | 0.00 | 0.00 |  | 0.02 | 0.02 |  | 0.01 | 0.01 |  | 0.01 | 0.01 |  | 0.03 | 0.02 |  | 0.00 | 0.00 |  | 0.00 | 0.00 |
|  | 30-<40 | 0.16 | 0.08 |  | 0.18 | 0.08 |  | 0.33 | 0.10 |  | 0.49 | 0.12 |  | 0.31 | 0.10 |  | 0.21 | 0.09 |  | 0.13 | 0.08 |
|  | 40-<50 | 0.20 | 0.09 |  | 0.63 | 0.16 |  | 0.58 | 0.15 |  | 0.56 | 0.14 |  | 0.97 | 0.17 |  | 0.77 | 0.15 |  | 0.64 | 0.15 |
|  | 50-<60 | 0.35 | 0.13 |  | 1.07 | 0.23 |  | 1.26 | 0.23 |  | 1.70 | 0.27 |  | 1.47 | 0.25 |  | 1.85 | 0.26 |  | 2.53 | 0.32 |
|  | 60-<70 | 0.73 | 0.23 |  | 1.41 | 0.29 |  | 1.68 | 0.32 |  | 2.02 | 0.34 |  | 2.21 | 0.33 |  | 3.57 | 0.41 |  | 3.20 | 0.43 |
|  | 70-<80 | 1.77 | 0.42 |  | 3.27 | 0.62 |  | 2.24 | 0.49 |  | 3.46 | 0.55 |  | 3.90 | 0.56 |  | 3.76 | 0.53 |  | 2.66 | 0.46 |
|  | 80+ | 2.92 | 0.92 |  | 3.58 | 0.96 |  | 4.28 | 1.01 |  | 2.27 | 0.76 |  | 5.40 | 1.04 |  | 4.94 | 0.89 |  | 6.51 | 1.06 |
| Female | 0-<30 | 0.00 | 0.00 |  | 0.01 | 0.01 |  | 0.01 | 0.01 |  | 0.00 | 0.00 |  | 0.01 | 0.01 |  | 0.03 | 0.02 |  | 0.02 | 0.02 |
|  | 30-<40 | 0.20 | 0.09 |  | 0.40 | 0.12 |  | 0.34 | 0.10 |  | 0.44 | 0.11 |  | 0.28 | 0.09 |  | 0.38 | 0.12 |  | 0.40 | 0.13 |
|  | 40-<50 | 0.49 | 0.14 |  | 0.40 | 0.13 |  | 1.25 | 0.22 |  | 1.85 | 0.26 |  | 1.64 | 0.23 |  | 1.88 | 0.23 |  | 1.17 | 0.21 |
|  | 50-<60 | 1.58 | 0.27 |  | 1.13 | 0.23 |  | 2.52 | 0.33 |  | 2.38 | 0.31 |  | 2.87 | 0.34 |  | 3.47 | 0.35 |  | 4.74 | 0.43 |
|  | 60-<70 | 2.38 | 0.34 |  | 3.12 | 0.37 |  | 3.54 | 0.42 |  | 3.14 | 0.40 |  | 3.42 | 0.39 |  | 5.10 | 0.46 |  | 5.15 | 0.52 |
|  | 70-<80 | 4.17 | 0.48 |  | 5.03 | 0.56 |  | 5.08 | 0.54 |  | 5.80 | 0.55 |  | 4.47 | 0.51 |  | 4.61 | 0.52 |  | 5.57 | 0.59 |
|  | 80+ | 4.82 | 0.75 |  | 7.23 | 0.86 |  | 5.78 | 0.73 |  | 7.92 | 0.87 |  | 7.81 | 0.79 |  | 6.78 | 0.70 |  | 6.76 | 0.77 |

SE: Standard error
